# Both Constitutive and Infection-Responsive Secondary Metabolites Linked to Resistance against *Austropuccinia psidii* (Myrtle Rust) in *Melaleuca quinquenervia*

**DOI:** 10.3390/microorganisms10020383

**Published:** 2022-02-07

**Authors:** Michelle C. Moffitt, Johanna Wong-Bajracharya, Louise S. Shuey, Robert F. Park, Geoff S. Pegg, Jonathan M. Plett

**Affiliations:** 1 School of Science, Western Sydney University, Campbelltown, NSW 2560, Australia; 2 Hawkesbury Institute for the Environment, Western Sydney University, Richmond, NSW 2753, Australia; johanna.wong@dpi.nsw.gov.au (J.W.-B.); j.plett@westernsydney.edu.au (J.M.P.); 3 New South Wales Department of Primary Industries, Elizabeth Macarthur Agricultural Institute, Menangle, NSW 2568, Australia; 4 Department of Agriculture and Fisheries, Queensland Government, Brisbane, QLD 4102, Australia; Louise.Shuey@daf.qld.gov.au (L.S.S.); Geoff.Pegg@daf.qld.gov.au (G.S.P.); 5 The Plant Breeding Institute, School of Life and Environmental Sciences, The University of Sydney, Sydney, NSW 2006, Australia; robert.park@sydney.edu.au

**Keywords:** *Austropuccinia psidii*, *Melaleuca quinquenervia*, myrtle rust, metabolomics, flavonoids, terpenoids

## Abstract

*Austropuccinia psidii* is a fungal plant pathogen that infects species within the Myrtaceae, causing the disease myrtle rust. Myrtle rust is causing declines in populations within natural and managed ecosystems and is expected to result in species extinctions. Despite this, variation in response to *A. psidii* exist within some species, from complete susceptibility to resistance that prevents or limits infection by the pathogen. Untargeted metabolomics using Ultra Performance Liquid Chromatography with Ion Mobility followed by analysis using MetaboAnalyst 3.0, was used to explore the chemical defence profiles of resistant, hypersensitive and susceptible phenotypes within *Melaleuca quinquenervia* during the early stages of *A. psidii* infection. We were able to identify three separate pools of secondary metabolites: (i) metabolites classified structurally as flavonoids that were naturally higher in the leaves of resistant individuals prior to infection, (ii) organoheterocyclic and carbohydrate-related metabolites that varied with the level of host resistance post-infection, and (iii) metabolites from the terpenoid pathways that were responsive to disease progression regardless of resistance phenotype suggesting that these play a minimal role in disease resistance during the early stages of colonization of this species. Based on the classes of these secondary metabolites, our results provide an improved understanding of key pathways that could be linked more generally to rust resistance with particular application within *Melaleuca*.

## 1. Introduction

The biotrophic plant pathogen *Austropuccinia psidii*, causal agent of the disease commonly known as myrtle rust, has a broad host range across many species within the Myrtaceae family. In Australasia, Myrtaceae predominate in most ecosystems, with more than 2250 species recorded [1]. They are also found in biological hotspots around the globe [2,3]. As such, *A. psidii* infection is a threat to many fragile ecosystems characterised by a high rate of endemism [4,5,6]. Further, as many species within this family are important globally due to their value in forestry, the essential oil industry as well as for cultural foods, this rust pathogen poses a significant threat economically [5,7]. To date, 539 Myrtaceae species are known to be vulnerable to *A. psidii* infection worldwide, including *Melaleuca quinquenervia* [8]. In 2020, the Australian Department of Agriculture, Water and the Environment listed two Myrtaceae species, *Rhodamnia rubescens* and *Rhodomyrtus psidioides* as critically endangered as a direct result of *A. psidii* infection in the wild [9]. Species belonging to *Rhodamnia, Rhodomyrtus, Gossia, Lenwebbia, Backhousia* and *Ristantia* are also severely affected by *A. psidii* and localised extinction of populations may be imminent within a generation [10]. In addition to decreased survival of Myrtaceae within ecosystems heavily impacted by *A. psidii*, increased transparency of the canopy resulting from dieback impacts overall species richness in the understory [11] and impacts regrowth following bushfires [12,13].

*M. quinquenervia* is a keystone species in the coastal wetlands and floodplains of northern New South Wales and Queensland regions of Australia and are important in maintaining water quality through nutrient filtering [14,15]. *M. quinquenervia* is also an important source of food and habitat for local animals in these regions, including vertebrates and invertebrates, as well as epiphytic plants [1].

Myrtle rust infects new shoots (including the leaves, petiole and stem, of leaf pairs 1–3) causing branch dieback, as well as flowers and fruits leading to infertility [10,16]. Once leaves become fully developed, ontogenic resistance occurs and as such infection occurs during the actively growing season [16]. *A. psidii* infection begins with germination of urediniospores around 6 h after coming into contact with plant tissues, followed by the formation of appressoria within 18 h [17]. Disease symptoms become visible on young leaves 3–5 days after inoculation followed by visible urediniospore pustule formation after approximately 12 days, although this latent period in the infection cycle is temperature dependent and can be as short as 7 days or be prolonged in colder temperatures [16]. There are a range of responses to infection on new shoots within some species of Myrtaceae, from strong resistance with no macroscopic symptoms to complete susceptibility [14,18]. The susceptible phenotype displays abundant spore formation on young leaves approximately 21 days after infection. Hypersensitive individuals display localised necrotic regions on young leaves indicating a rapid resistance response. Genomic approaches have been utilised to better understand pathways and genetic architecture associated with host resistance to *A. psidii* [19,20,21,22,23,24]. Gene expression analysis has indicated that early recognition of the pathogen within 24 h post-infection and activation of defence responses discriminate resistant individuals of *Eucalyptus grandis* and *Syzygium luehmannii* from susceptible individuals [21,23]. Other transcriptomic studies, including in *M. quinquenervia*, have highlighted differential expression of genes related to brassinosteroid signalling, G-type lectin receptor-like kinases, toll/interleukin-1 family proteins, and nucleotide-binding site leucine-rich repeat proteins as being common to resistant individuals [21,22,23,24,25]. In these species, the transcriptomic response of the plant during the early stages of infection appears to be critical in determining the level of host resistance.

Recently, untargeted metabolomics has shown promise in identifying disease resistance biomarkers present in a broad range of plants, including those found early in *Phytophthora* infection of tomatoes [26], *Phakopsora pachyrhizi* infection of soybean [27], and *Plasmopara viticola* defence response in a resistant grape variety [28]. This technique profiles all small molecules that are present within a biological sample at a given time. These molecules can include substrates, intermediates and products of metabolic pathways, as well as signalling molecules, hormones, and secondary metabolites. The benefit of metabolomics over other omics-based strategies of identifying disease resistance markers is that no genome sequence information is required, and the method can be applied in non-model systems. One of the drawbacks of this approach is that compound reference databases are still being developed and metabolite identification can be difficult. However, given their applications in other pathosystems, it is possible that untargeted metabolomics could be used to characterise the defence response by Myrtaceae to *A. psidii* infection. To date, targeted metabolomics has investigated the possibility of using terpenes as resistance markers [29,30,31], while untargeted metabolomics has been used to evaluate metabolic fluxes during the early stages of *A. psidii* infection in *E. grandis* [25]. Various attributes have been investigated in pre-formed resistance to *A. psidii* infection in Myrtaceae species. These include cuticular waxes [32] and terpene composition in *Eucalyptus* sp. and *M. quinquenervia* [29,31], while increasing terpene concentration in older leaves may be associated with ontogenic resistance [33]. These previous studies are only just beginning to inform our understanding of the mechanisms that have evolved within the Myrtaceae that enable certain individuals/species to resist infection by *A. psidii.*

The broad range of applications of metabolomics demonstrates that this methodology could be used in assessing mechanisms and identifying novel biomarkers of host resistance to *A. psidii*. *M. quinquenervia* represents an ideal host species to identify these biomarkers as there are individuals described previously as susceptible, hypersensitive and resistant to *A. psidii* infection [34]. By evaluating the response of different *M. quinquenervia* resistance phenotypes to *A. psidii*, we aimed to determine if a set of metabolites are involved in innate resistance to the disease and to understand what metabolic pathways are altered by the colonisation process.

## 2. Materials and Methods

### 2.1. Austropuccinia psidii Inoculation of Melaleuca quinquenervia

**Figure 1:**
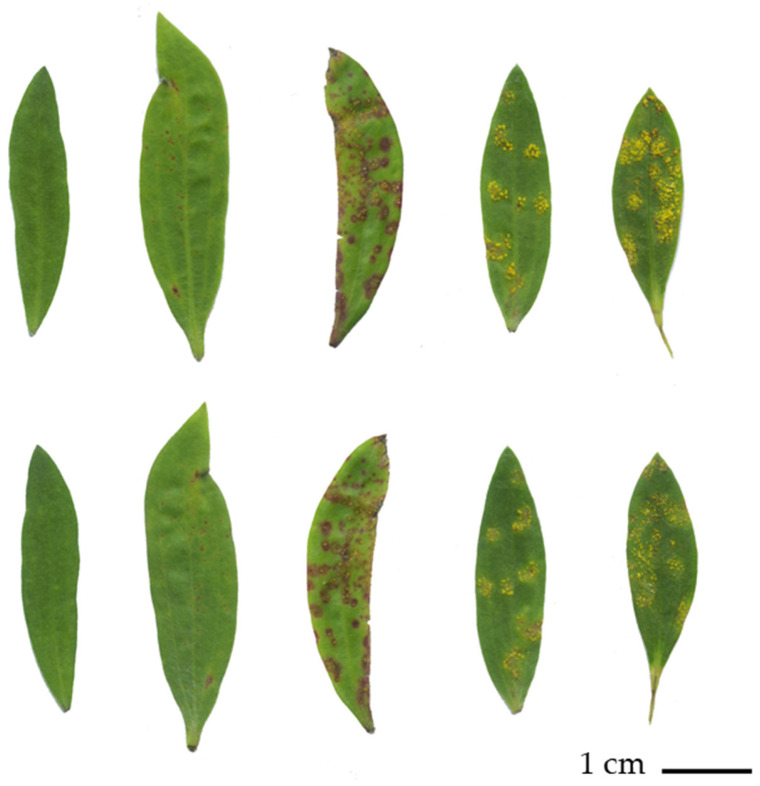
Images of *A. psidii* infection ratings of *M. quinquenervia* plants assessed in this study according to Pegg et al., 2018, based on the method developed by Junghans et al., 2003. The top row represents the adaxial view of the leaves while the bottom row represents the abaxial view. From left to right, leaf 1 represents rating (R) 1, the resistant phenotype, displaying no visible *A. psidii* pustules or necrotic legions. Leaf 2 represents R2, the hypersensitive phenotype, displaying necrotic legions but no visible pustules. Leaves 3–5 represent susceptibility R 3–5, displaying urediniospore pustules of increasing size. Plants demonstrating susceptibility R5 were selected for the susceptibility phenotype in this study.

A total of nine *M. quinquenervia* individuals of previously defined *A. psidii* infection phenotype (three each of resistant, hypersensitive and susceptible) were chosen for analysis. Plants were rated according to the disease ranking system proposed previously [14,35] and ranked as resistant (susceptibility rating 1, no visible *A. psidii* pustules or nectrotic legions), three hypersensitive (susceptibility rating 2, evidence of necrotic lesions but no visible *A. psidii* pustules) and three susceptible (susceptibility rating 5, large visible pustules) (Figure 1). Each plant was coppiced prior to infection to promote branching and the production of new leaves and subsequently inoculated with *A. psidii* at the Queensland Department of Agriculture and Fisheries. Spore collection and inoculation were as per Pegg et al. 2018; briefly, urediniospores, collected from infected *Syzygium jambos* trees, were suspended in a sterile distilled water solution to which the surfactant Tween 20 was added at a rate of two drops per 100 mL. The spore concentration in the suspension was assessed using a haemocytometer adjusted to 1 × 10^5^ spores/mL. Plants were inoculated using a fine mist spray (29 kPa pressure), generated by a compressor driven spray gun (Iwata Studio series 1/6 hp.; Gravity spray gun RG3). The upper and lower leaf surfaces of the seedlings were sprayed, ensuring all leaves were coated with a fine mist but run-off of the spore suspension was avoided. Once inoculated, seedlings were placed into a Controlled Environment Room (CER) set at 18 °C in the dark for 24 h.

Samples of leaves were collected prior to inoculation (i.e., 0 h), 24 h, 48 h post inoculation (hpi) and 5 days post inoculation (dpi). Six replicates of young leaves of the same plastochron index (to avoid ontogenic effects) from fresh growth on each plant were collected at each time point, weighed and stored at −80 °C prior to analysis.

### 2.2. Leaf Metabolite Extraction

The frozen leaf samples were weighed and immediately ground into powder by bead-beating in 200 µL extraction solvent (4:4:2 methanol:acetonitrile:deionised water) twice for 30 s each. The leaf:solvent mixture was then combined with 300 µL additional extraction solvent, and then vortexed to mix for 10 s. The leaf:solvent mixture was then placed in icy water and sonicated in a sonicating water bath for 25 min. Solid cellular material was removed by centrifugation at 18,000× *g* for 10 min. The supernatants were collected while avoiding undissolved particles and filtered through a 0.22 µm syringe filter. The resulting metabolite extracts were stored at −80 °C until metabolite profiling was performed.

### 2.3. Ultra Performance Liquid Chromatography High Definition Mass Spectrometry with Ion Mobility (UPLC HDMS^E^) Analysis

Leaf extracts were analysed on a Waters Acquity I-Class UPLC system and a Waters Synapt G2-Si HDMS with a Waters UniSpray Ionisation source at the Western Sydney University Mass Spectrometry facility. The metabolites were separated on a Waters ACQUITY UPLC HSS T3 1.8 µm 100 × 2.1 mm Column at 35 °C. The injection volume was 2 μL. The mobile phases were A (Water + 0.1% Formic Acid) and B (Acetonitrile + 0.1% Formic Acid). The chromatographic flow rate was 0.5 mL/min with a 9 min gradient, with mobile phase A held at 99% for 1 min, decreased to 85% over 1 min, decreased to 50% over 2 min, decreased to 5% over 2 min and increased to 99% over 2 min. Leucine Enkephalin Lockspray solution (Waters, 1 ng/mL) was used as a standard.

Data acquisition was performed with ion mobility separation followed by mass fragmentation and high-resolution mass analysis. The mass range of metabolites acquired was 50–1200 *m*/*z*, the scan time was 0.2 s and the elevated energy transfer collision voltage was 20–50 eV. For this experiment, the instrument was run in positive ionisation mode with the following settings: Capillary: 0.5 kV, source temperature: 120 °C, sampling cone: 30 V, source offset: 80 V, desolvation temperature: 500 °C, desolvation gas flow: 800 L/h, cone gas flow: 20 L/h.

### 2.4. Statistical and Molecular Analysis

The compound measurement table containing the feature spectral information and peak intensity of all samples (including quality-control samples and solvent blanks) was exported from the Progenesis QI for metabolomics (version 2.4) and further analyses were performed using R (version 3.6.3). The data table was scaled by leaf weight and low intensity peaks (intensity < 500) were removed after blank subtraction. The data table was then imported into MetaboAnalystR (version 3.0.3) [36]. To improve the power of downstream statistical testing, features that are missing in >50% samples or near-constant across all samples were filtered based on interquartile range. Additionally, outliers were removed after visual identification using principal component analysis (PCA). The data table was log-transformed before two-way analysis of variance (ANOVA) with multiple comparisons correction (false discovery rate). The significant features with *p* < 0.05 were selected for further clustering analyses. Hierarchical clustering and k-means clustering were performed using R (version 3.6.3). The log-transformed data table was also used for permutational multivariate analysis of variance (PERMANOVA) with the pairwise Adonis wrapper (https://github.com/pmartinezarbizu/pairwiseAdonis accessed on 10 December 2021) based on the ‘adonis’ function of the vegan package (version 2.5-6) on R. Progenesis QI was used to structurally classify the significant metabolites identified by the two-way ANOVA and clustering analyses. Based on the identities provided by the Chemical Entities of Biological Interest (ChEBI), metabolite enrichment analysis was performed on the MetaboAnalyst web platform (https://www.metaboanalyst.ca/ accessed on 10 December 2021). Metabolomics data will be made publicly available following acceptance of the manuscript for publication.

## 3. Results

### 3.1. Metabolomic Response to Myrtle Rust Infection in M. quinquenervia

**Figure 2:**
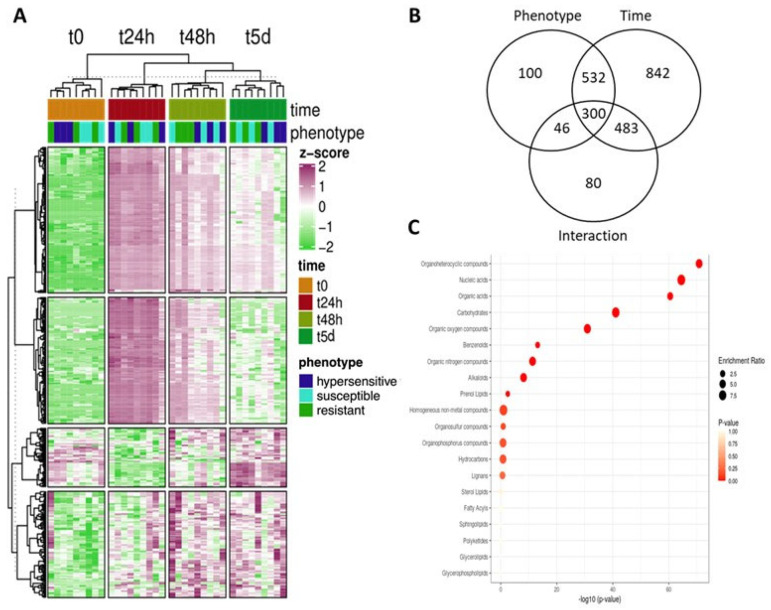
*Austropuccinia psidii* trigged changes in the abundance of molecular feature recovered from *Melaleuca quinqueneriva* leaves is a function of host resistance phenotype and stage of infection. (**A**) Heatmap showing the scaled peak intensity of the top-500 significant molecular features (MFs; ranked by adjusted p calculated with 2-way ANOVA). The heatmap is clustered by Euclidean distance and clustered with Ward’s minimum variance method for both the columns and rows. Purple and green indicate the increase and/or reduction in abundance, respectively, of each significant molecular feature (rows) of each sample (columns). (**B**) Venn diagram showing the number of MFs significantly changed in terms of abundance under the influence of phenotype, time post-inoculation and the interaction of both phenotype and time. Two-way ANOVA with multiple-comparison adjustment using the false discovery rate approach was performed to identify the significant metabolites (adjusted-*p* < 0.05) influenced by each factor and their interaction. (**C**) Enrichment analysis of MF classification of the significant metabolites influenced by phenotype-only (adjusted *p* < 0.05, two-way ANOVA with FDR adjustment). The *x*-axis and colour of the points indicate the significant value of the enrichment set, whereby the size of the points indicate the enrichment ratio generated by metabolite enrichment analysis.

*M. quinquenervia* plants belonging to one of three phenotypes (resistant, hypersensitive and susceptible) were analysed for their metabolomic responses to the early stages of *A. psidii* infection. Six leaves of the same plastochron index were sampled prior to inoculation (0 h) and 24 h, 48 h and 5 days after inoculation (total 216 leaves). The metabolome of the leaves at each time point was analysed by LC-MS and after the removal of outliers, five biological replicates were analysed and a total of 11,276 molecular features (MFs) were identified. Analysis of the leaf metabolomes identified that the resistance phenotype of the individual, time post-inoculation and the interaction of these two factors were all significant as determined by permutational multivariate analysis of variance (PERMANOVA; Table 1). Amongst these MFs, we found 2383 MFs have significant difference in abundance under influence of time post infection, phenotype of the plants or the interaction of both (*p* < 0.05; Top 500 MFs abundance plotted in Figure 2A; Supplemental Table S1). The abundance of 978 and 2157 MFs were influenced by the phenotype of the plants and the time after inoculation, respectively, while 909 MFs were influenced by the interaction of phenotype by time post infection (Figure 2B).

### 3.2. Organoheterocyclic Compounds Significantly Enriched According to Resistance Phenotype Regardless of Time Post Infection

We further analysed the metabolomics profile of resistant, hypersensitive and susceptible phenotypes of *M. quinquenervia* in response to infection. Although hierarchical clustering did not clearly separate MF profiles of leaves following infection from different phenotypes, both PERMANOVA and ANOVA results support that there is significant variation in the leaf MF profiles from *M. quinquenervia* of different resistance phenotypes. In an effort to identify the MFs that distinguish susceptibility of *M. quinquenervia* to *A. psidii*, enrichment analysis was performed on the metabolite responses that were significantly influenced by phenotype, irrespective of time (ANOVA adjusted-*p* < 0.05). Based on the MFs for which structural predictions were made by Progenesis using the CHEHBI database, organoheterocyclic compounds, nucleic acids, organic acids and carbohydrates appeared to be the most enriched metabolite sets that differentiated these phenotypes following application of the pathogen (Figure 2C; Supplemental Table S2).

### 3.3. Few Metabolites Are Significantly Associated in a Resistance Phenotype × Time Post-Infection Manner

To establish the chemical classes that are important in differentiating the phenotypes × time, metabolite enrichment analysis was performed on 24 and 48 hpi samples. We found that only 19 MFs were unique to resistant x time, one was unique to the hypersensitive phenotype x time and three were unique to the susceptible phenotype × time interaction. Of these MFs, the identities of only seven could be structurally predicted based on mass spectrometry fragmentation patterns (Table 2). Using PubChem compound summaries, we were able to assign probable compound super/subclasses or pathways to these seven metabolites. Included in these MFs were a range of defence-related classes including fatty acyl glycosides, hormone-related MFs (e.g., sphingolipid/ceramide, phosphatidylglycerol), a compound structurally similar to quorum sensing molecules, as well as a pyrethrin. 

### 3.4. Terpenoids Alter with Time Post-Infection but Not with Resistance Phenotype

**Figure 3:**
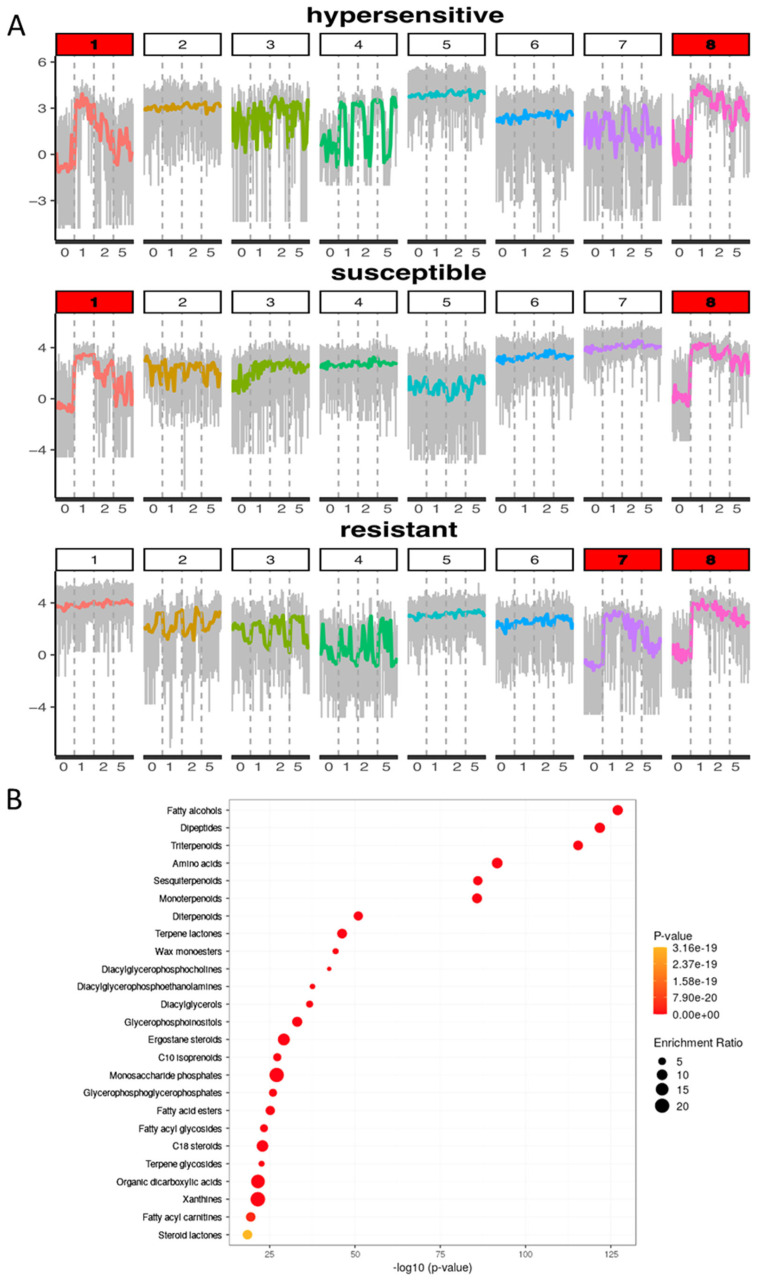
Enrichment of fatty alcohols, dipeptides and triterpenoids is a common response of all phenotypes in *Melaleuca quinquenervia* leaves 24 and 48 h after *Austropuccinia psidii* inoculation (**A**) K-means clustering separates metabolites into groups with different temporal shifts in abundance. Metabolite responses prior to infection (0), at 24 hpi (1), 48 hpi (2), and five days post infection (5). The line in each plot indicates the average log (peak intensity) of the molecular features (y-axis) across the time-series (x-axis; number of days post-inoculation). The metabolites in the key clusters highlighted in red have an increased abundance at the 24 h and 48 h after infection time points. (**B**) Enrichment graphic of molecular features recovered from *M. quinquenervia* leaves that display a time-only interaction. The x-axis and colour of the points indicate the significant value of the enrichment set, whereby the size of the points indicate the enrichment ratio generated by metabolite enrichment analysis.

Time post-inoculation, regardless of resistance phenotype, appears to be the most influential factor with respect to changes within the metabolome. While some of these responses may be associated with leaf aging, as we only used one timepoint for an un-infected control, these results show that *A. psidii* infection is able to trigger major metabolite responses in *M. quinquenervia* leaves. We further examined the temporal changes of these significant MFs identified with analysis of variance (ANOVA). Through hierarchical clustering analysis, we found that a majority of these significant MFs were enriched shortly after *A. psidii* infection (24 or 48 h after inoculation) and gradually reduced at 5 days after inoculation (Figure 2A). Based on these results, we used a *k*-means clustering approach for each phenotype to group the metabolites based on the pattern of temporal changes. For each phenotype, we identified two clusters of inducible metabolites at 24 and 48 h post inoculation (Figure 3A; denoted by red title). Of the 283 MFs identified within these six clusters, a majority are shared by the different phenotypes. The structural classes of these inducible MFs are terpenoids, fatty alcohols, and dipeptides, and included intermediates of phytohormone pathways (Figure 3B; Supplemental Table S3).

### 3.5. Pre-Infection Enrichment of Flavonoid Compounds Found in Resistant Individuals

**Figure 4:**
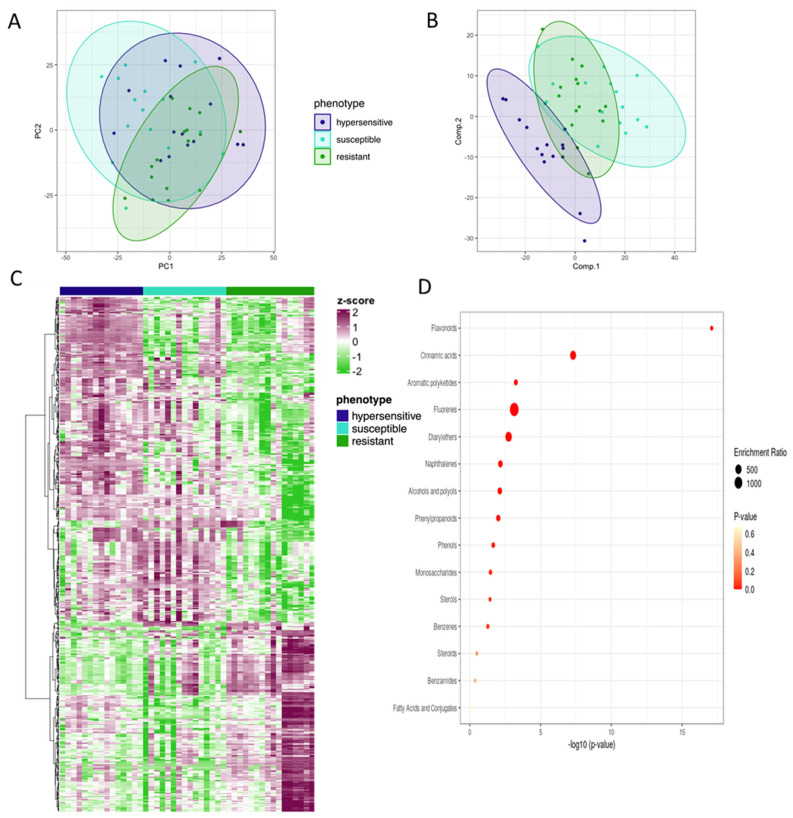
Leaf molecular feature abundance differentiates resistance phenotype prior to *Austropuccinia psidii* inoculation. (**A**) PCA or all samples; (**B**) PLS-DA of all samples; (**C**) Heatmap of scaled peak intensity of the significant molecular features (ranked by adjusted p calculated with 2-way ANOVA). The heatmap is clustered by Euclidean distance and clustered with Ward’s minimum variance method for both the columns and rows. Purple and green indicate the enrichment and reduction, respectively, of each significant molecular feature (rows) of each sample (columns). (**D**) Flavonoid compounds are the predominant chemical class of metabolites that differ between different *M. quinquenervia* disease phenotypes before inoculation with *A. psidii*. The x-axis and colour of the points indicate the significant value of the enrichment set, whereby the size of the points indicate the enrichment ratio generated by metabolite enrichment analysis.

To establish if metabolites are important in defining *M. quinquenervia* resistance to *A. psidii* before infection, uninfected leaf samples of the same plastochron index sampled at the same time were analysed by principal component analysis; no significant grouping was found according to phenotype (Figure 4A). Subsequently, PLS-DA and ANOVA analysis was applied to identify if any MFs could significantly separate the three phenotypes (Figure 4B). The heatmap of the top MFs (ranked by the ANOVA FDR value) shows that resistant individuals were characterised by lower relative abundances of the majority of the MFs identified (Figure 4C; Supplemental Table S4). To determine the chemical classes of MFs that are associated with resistance to *A. psidii* before infection, metabolite enrichment analysis was performed. The MFs enriched in the resistant phenotype that were structurally predicted using Progenesis included the chemical classes of flavonoids, cinnamic acids and polyketides (Figure 4D; Supplemental Table S5).

## 4. Discussion

*Melaleuca quinquenervia* is a suitable species of the Myrtaceae for improving our understanding of the mechanisms related to resistance to *A. psidii* as individuals within this species display a range of susceptibility from complete resistance to complete susceptibility. We sought to use an untargeted metabolomics approach to identify global changes to a broad spectrum of known and unknown metabolites following *A. psidii* infection and to predict the metabolic basis of infection phenotype. Our findings demonstrate that regardless of rust response phenotype, all individuals tested exhibited temporal changes in the metabolome in response to pathogen challenge. Metabolites found to be associated with pathogen defence in other plant species were found to be more enriched in resistant *M. quinquenervia* individuals. Furthermore, our analyses suggest that pre-existing differences in metabolic profiles in the leaves of *M. quinquenervia* prior to infection may serve as an addition means by which we can predict resistance phenotypes.

### 4.1. Melaleuca quinquenervia Synthesises MFs in Response to Pathogen Challenge That Are Independent of Infection Phenotype

Plants are universally responsive to colonisation by microbes, regardless of the degree of damage they may cause [37,38]. Following inoculation, *A. psidii* urediniospores germinate and form appressoria within the first 18 h [17]. Previous studies in *S. luehmannii* and *E. grandis* demonstrated that the host is very responsive transcriptomically at this stage [21,23,24]. In *S. luehmannii,* the greatest host response was observed 48 h after pathogen challenge, with pathways including a number of secondary metabolic pathways (e.g., flavonoid metabolic enzymes, cell wall active enzymes) [21]. Brassinosteroid signalling was also observed in E. grandis at the same time point [24]. Metabolically, Sekiya et al. (2020) found that the number of differentially regulated MFs of *E. grandis* during infection stayed consistent, or increased slightly in number, throughout the a range of timepoints through 24 h post inoculation, while in the wheat:*Puccinia triticina* pathosystem, differential enzyme responses were observed at 48 h post inoculation [39]. Similarly, in the soybean:*Phakopsora pachyrhizi* interaction, changes in host metabolome increased through 72 h post infection [40]. Our results show that in *M. quinquenervia*, a metabolomic response was triggered in all plants upon infection, with a large overlap in the MFs regulated between individual plants tested. The metabolomic changes were greatest at 24 hpi, decreasing at 48 hpi and approaching pre-infection levels by day 5. This indicates that a strong chemical defence response to the pathogen is triggered in *M. quinquenervia*, regardless of phenotype, and that this metabolic response was faster and greater than documented in other model systems considered here [21], while some MF responses may have been resultant of leaf age [25]. Interestingly, a range of terpenoids were among the induced metabolites that did not vary by resistance phenotype. Several studies have considered the role played by terpenoids during host interaction with biotrophic pathogens, however, a clear role of these molecules has yet to be clearly established as some terpenes are associated with disease resistance [33,41,42], while others with susceptibility [30,43,44,45]. For example, in grapevine challenged with the biotrophic oomycete *Plasmopara viticola,* a phenotype by time-relationship was found for terpenes in resistant cultivars only where pathogen growth and reproduction were significantly repressed in plant hosts producing higher levels of terpenoids [46]. Our results are, therefore, different from these previous studies and suggest that in *M. quinquenervia* terpenoids may not be the most significant class of compound associated with defence against *A. psidii*. Despite this, our data suggest that the host is responsive metabolically to challenge by rust infection, and that small changes to host metabolism following infection must be considered as they may be deterministic in establishing the difference in between what defines a susceptible versus resistant phenotype.

### 4.2. Melaleuca Disease Resistance Involves MFs That Are Both Phenotype-Specific and Responsive to Phenotype × Time

The degree to which a host responds metabolically to colonisation by a pathogenic microbe can determine the disease resistance phenotype. Similarly, an interactive effect between host phenotype and the stage of disease development (i.e., phenotype × time interactive effect; [47]) may play a role in determining the resistance phenotype in a plant. Our study found evidence for metabolites tied to the level of *M. quinquenervia* resistance to *A. psidii* that were both phenotype-specific as well as associated in a phenotype × time manner. In the former category, we found that organoheterocyclic compounds, metabolites associated with nucleic acids, organic acids and carbohydrates, were all enriched in resistant individuals regardless of time. The metabolite superclass of organoheterocyclic compounds, including subclasses such as carbazole, porphyrin, and butanolide, are almost universally associated with anti-microbial or antioxidant activities [48,49,50]. Therefore, induction of these compounds by the onset of fungal colonisation within resistant individuals is likely to enhance defence against the pathogen. Compound classes such as carbohydrates, and their putative role in host resistance or susceptibility, are more difficult to interpret. Past work with leaf rust pathogens suggests that carbohydrate rich leaves are more susceptible to infection as they are rich in growth limiting nutrients for the pathogen [51]. This hypothesis, however, is not supported by more recent work that found leaf nutritional status had no impact on the development of *Melampsora laricipopulina* in the leaves of *Populus deltoides* × *P. nigra*, but rather that secondary metabolism differences between leaf ages were the driver of disease outcomes [52]. As we only assayed leaves of consistent age within our analyses, it is likely that differences in carbohydrates are tied to phenotypic variation between individuals and future work will be important to understand the roles of these metabolites in host resistance.

Of the metabolites that exhibited a link to disease resistance in a phenotype by time interaction, structural-based MF prediction identified a number of metabolites putatively associated with phytohormone defence pathways, with two MFs linked to salicylic acid biosynthesis and signalling (Table 2; [53,54]. SA has been shown to have a primary role in resistance to rust pathogens including *M. larici-populina:Populus* [55], *Puccinia triticina* [39] and *Uromyces* spp. [56]. Recent work suggesting that SA is involved in localised repression of haustorial formation [57] would explain why we do not observe physical damage within resistant lines of *M. quinquenervia*. A quorum sensing molecule, methyl 3-hydroxypalmitate, was also identified as increasing in concentration with time only in resistant individuals. Quorum sensing compounds have previously been characterised as being pathogen-produced (e.g., *Rolstonia solanacearum;* [54]) and necessary for the progression of disease. The discovery of this MF was unanticipated because it was induced in resistant *M. quinquenervia* individuals without visible disease symptoms. It is possible that the compound was host-produced as other research suggests that quorum sensing-like molecules can be produced by plants to recruit biocontrol bacteria [58]. Therefore, in addition to considering the specific MFs produced in plants during disease challenge, the timing of their production must also be considered as different MFs will have roles in host immunity by affecting development of the pathogen from spore germination through to reproduction.

### 4.3. Basal Metabolic Processes May Contribute to Myrtle Rust Resistance

In a range of pathosystems, research has sought to predict disease resistance ratings of plant varieties based on basal metabolism in the absence of the disease-causing microbe. These research strategies are based on the hypothesis that steady states of different physical or chemical defence compounds may be naturally higher in some individuals, thereby affording them greater protection against the initial stages of disease. Physical barriers associated with cuticular waxes enriched in hexadecenoic acid relate positively to increased rust resistance in *E. grandis* and *Eucalyptus phaeotricha* [32], while elevated levels of limonene have also been associated with rust resistance [33]. We also detected consistent differences in the metabolomic profile of the individual *M. quinquenervia* plants prior to infection that significantly predicted disease resistance ratings. In particular, flavonoids were enriched in the resistant plants. A number of studies have highlighted that flavonoids may inhibit disease progression by inhibiting germination of fungal spores or fungal growth [59,60], while others have shown an opposing role [61,62]. Other plant studies have shown that flavonoids are important in the resistance of plants to biotic stress, for example potato leaves resistant to late blight were enriched with higher concentrations of some flavonoids [63]. In resistant *E. grandis* flavonoids are increased in abundance in resistant lines 24 hpi, which was further supported by increased abundance of phenylpropanoid and flavonoid biosynthetic proteins [25]. Similarly, transcriptomic analysis in *Syzygium luehmannii* identified elevated expression levels of the enzyme phenylalanine ammonia lyase in resistant plants, which supplies the precursor for flavonoid metabolism [21]. Therefore, while the exact flavonoids found in our work are predictions only, flavonoid molecules present within certain *M. quinquenervia* individuals appear to be major determinants in myrtle rust resistance.

## 5. Conclusions

Altogether, our research suggests that the resistance to *A. psidii* in *M. quinquenervia* is mediated in part by two separate pools of metabolites: those that are basally produced in leaves and those that are pathogen inducible. The similarity in metabolite classes found to predict myrtle rust resistance in *M. quinquenervia* to those found to predict resistance to biotrophic pathogens in a broad range of plants provide promise that these MFs could be resistance biomarkers that are not just relevant within *Melaleuca*, but also to other Myrtaceae. Further studies are required, therefore, to determine if this is the case and, where a consistent subset of biomarkers is identified, adapted to enable screening for resistant germplasm in nursery breeding programs to protect the biodiversity in this ecologically, culturally, and economically important plant family.

## Figures and Tables

**Table 1:** Summary of pairwise permanova analysis.

Comparison	Factors	Degree of Freedom	R2	*p*-Value
Hypersensitive_vs_Resistant	Phenotype	1	0.04669	0.001 ***
	Time	3	0.34827	0.001 ***
	Phenotype × Time	3	0.02854	0.015 *
Hypersensitive_vs_Susceptible	Phenotype	1	0.02803	0.001 ***
	Time	3	0.37494	0.001 ***
	Phenotype × Time	3	0.02648	0.032 *
Resistant_vs_Susceptible	Phenotype	1	0.05663	0.001 ***
	Time	3	0.36828	0.001 ***
	Phenotype × Time	3	0.03643	0.002 **

* *p*-value ≤ 0.05; ** *p*-value ≤ 0.01; *** *p*-value ≤ 0.001.

**Table 2:** Structural prediction of molecular features enriched in the resistant phenotype 24 and 48 h post inoculation.

*m/z*	Predicted Structure	Superclass/Pathway	Identification Score
367.1960321	(2R,6x)-7-Methyl-3-methylene-1,2,6,7-octanetetrol 2-glucoside	Fatty acyl glycoside	38.7
624.5280082	N-icosanoyl-15-methylhexadecasphing-4-enine	Sphingolipid/ceramideSA Biosynthesis	35.8
791.4805652	1-oleoyl-2-[(3E)-hexadecenoyl]-sn-glycero-3-phosphoglycerol	Phosphatidylglycerol Activation of JA signalling	37.4
636.5160247	methyl 3-hydroxypalmitate	Quorum sensing	38.4
657.2921888	coproporphyrinogen III(4-)	Salicylic acid pathway	37.7
623.3873252	lutein 5,6-epoxide	Carotenoid	32.4
609.8434753	cis-3-(2,2-dibromovinyl)-2,2-dimethylcyclopropanecarboxylic acid	Pyrethrin	23.8

## Data Availability

Links to publicly available data will be provided upon acceptance of the manuscript for publication.

## Supplementary Materials

The following supporting information can be downloaded at: www.mdpi.com/article/10.3390/microorganisms10020383/s1, Table S1: The top 500 molecular features that were significantly changed in abundance during the experiment, Table S2: Enriched molecular features that differentiated the phenotypes during early stages of infection with *Austropuccinia psidii,* Table S3: Structural prediction of molecular features enriched in *Melaleuca quinquenervia* leaves during the early stages of infection by *Austropuccinia psidii*, Table S5: Structure predictions of molecular features enriched in the resistant *Melaleuca quinquenervia* leaves before infection with *Austropuccinia psidii.*
